# Work related musculoskeletal disorders amongst therapists in physically demanding roles: qualitative analysis of risk factors and strategies for prevention

**DOI:** 10.1186/1471-2474-12-24

**Published:** 2011-01-25

**Authors:** Leanne Passier, Steven McPhail

**Affiliations:** 1 Physiotherapy Department, Princess Alexandra Hospital, Ipswich Road, Brisbane, Australia; 2 Centre for Functioning and Health Research, Buranda Plaza, Corner Ipswich Road and Cornwall Street, Buranda, Brisbane, Australia; 3 Queensland University of Technology, School of Public Health and Institute of Health and Biomedical Innovation, Kelvin Grove, Brisbane, Australia

## Abstract

**Background:**

Physiotherapy and occupational therapy are two professions at high risk of work related musculoskeletal disorders (WRMD). This investigation aimed to identify risk factors for WRMD as perceived by the health professionals working in these roles (Aim 1), as well as current and future strategies they perceive will allow them to continue to work in physically demanding clinical roles (Aim 2).

**Methods:**

A two phase exploratory investigation was undertaken. The first phase included a survey administered via a web based platform with qualitative open response items. The second phase involved four focus group sessions which explored topics obtained from the survey. Thematic analysis of qualitative data from the survey and focus groups was undertaken.

**Results:**

Overall 112 (34.3%) of invited health professionals completed the survey; 66 (58.9%) were physiotherapists and 46 (41.1%) were occupational therapists. Twenty-four health professionals participated in one of four focus groups. The risk factors most frequently perceived by health professionals included: work postures and movements, lifting or carrying, patient related factors and repetitive tasks. The six primary themes for strategies to allow therapists to continue to work in physically demanding clinical roles included: organisational strategies, workload or work allocation, work practices, work environment and equipment, physical condition and capacity, and education and training.

**Conclusions:**

Risk factors as well as current and potential strategies for reducing WRMD amongst these health professionals working in clinically demanding roles have been identified and discussed. Further investigation regarding the relative effectiveness of these strategies is warranted.

## Background

High rates of employee injury within the health care industry are well documented[1-16]. Previous reports regarding the incidence of work related musculoskeletal disorders (WRMD) indicate that physiotherapy (also known as physical therapy) and occupational therapy are two professions that are at high risk [5,11,14,16]. Studies amongst physiotherapists (PT) have revealed as many as 91% experience WRMD during their career[5] with recurrence rates of up to 88%[16]. It has also been reported that 80% of PT experience symptoms in at least one body area over a 12 month period [5]. One in six PT have been reported to change their area of specialty or leave the profession as a result of pain or injury[5].

The physically demanding nature of work tasks and clinical demands are believed to contribute to this high incidence of WRMD amongst therapists [8,17-19]. This physically demanding nature has been observed to result in the adoption of awkward postures, such as bending and twisting [19]. Additionally time pressures associated with the clinical environments can result in therapists not taking break entitlements in order to meet patient treatment demand [19,20].

A similar prevalence of WRMD amongst occupational therapists (OT) has been extrapolated from a systematic review of the literature for other health disciplines [3]. A recent report provided the first preliminary empirical evidence specific to OT, in which a convenience sample reported a career incidence of 80% and twelve month incidence of 63% for WRMD [11]. These conditions may be associated with considerable personal and financial cost to individuals and have significant impact on the career path and retention of health professionals [3,5,11].

It is imperative that strategies to effectively prevent and manage WRMD are sought in order to reduce the burden on health professionals and sustain a viable workforce. Prevention of WRMD in nursing professionals has been extensively investigated and whilst there are similarities in clinical environment, patient types [9], and some overlap in patient handling there is a need to explore strategies specific to PT and OT due to the different nature of their clinical tasks [21,22]. Recommendations have been made around legislative requirements[23] and policy statements issued by relevant professional bodies [24,25]. Some studies[5,8,9,16,26,27] have described strategies used by PT or OT in response to WRMD, however, there have been few investigations of strategies currently being used by these health professionals to prevent WRMD [5,8]. Only one study of hand therapists[26] (PT and OT) has investigated PT or OT views regarding strategies that could be implemented to further reduce risk of WRMD.

An exploratory investigation of these issues amongst PT and OT clinicians from across a range of clinical settings was required to inform future injury prevention efforts to help retain health professionals in clinical roles. Due to the overlap between patient related factors and many clinical tasks completed by OT and PT clinicians, it was considered worthwhile to investigate risk factors and strategies to prevent WRMD amongst both OT and PT professionals.

In order to address this need, this investigation explored issues of WRMD as perceived by the health professionals working in these roles. The first aim was to identify the risks perceived by PT and OT as most likely to limit their capacity to continue working in physically demanding roles. The second aim was to identify strategies these health professionals are currently using, or believe could be used in the future, to enable them to continue to working in a physically demanding clinical environment. Due to the exploratory nature of this investigation there were no specific hypotheses regarding the likely similarity or differences in responses between the two professional groups.

## Methods

### Design

This investigation consisted of two phases of data collection. The first phase was a survey administered via a web based platform with qualitative open response items. The second phase involved focus group sessions which explored in more detail topics obtained from the survey.

### Participants and setting

A total of 327 health professionals including PT (186) and OT (141) across three health districts in Queensland, Australia were invited to participate in the survey. These three districts were selected to enable the inclusion of participants from a broad range of clinical settings. Health service settings for these professionals included major metropolitan hospitals providing acute, rehabilitation, specialist and outpatient services, regional hospitals and community, domiciliary, residential and mental health services. All PTs and OTs at participating sites were invited to take part in this investigation. Focus group participants included a convenience sample (n = 24) of survey respondents who were able to attend one of the four scheduled focus group sessions. The four focus groups were held at different geographical locations across the participating districts.

### Materials

The custom designed, web based survey consisted of four sections. The first section contained questions about demographic information including age, clinical experience and employment setting. The second section asked respondents to highlight perceived risk factors that may prevent them from continuing to work in physically demanding roles. The third section asked respondents to identify strategies that they currently employ to enable them to continue to work in physically demanding roles. The fourth section investigated potential strategies that could be implemented in the future to enable these health professionals to continue to work in physically demanding roles.

### Procedure

#### Survey

An email was distributed to all PT and OT staff at participating facilities containing a hyperlink to the web based survey platform which allowed respondents to complete the survey at their convenience and submit it online. The email was distributed to all staff email addresses within the relevant departments. Respondents also had the option to complete the survey in hard copy and return it via mail or facsimile. The survey was advertised widely across participating departments via email, notice boards, staff meetings, and site visits in order to maximise response rate. An email reminder was sent out one week before the closure of the 3 week data collection period. Approximately 20 minutes was required for participants to complete the survey. No personal identifying information was attached to responses.

#### Focus groups

Focus groups explored the top four most frequently perceived risk factors identified in the second section of the survey as likely to limit the health professional's capacity to continue working in physically demanding roles. Risk factors were used to stimulate discussion of current and potential strategies to minimise or eliminate these risks. Focus group discussions were transcribed to permit subsequent analysis.

### Analysis

Demographic information from the survey was tabulated (Table 1).

**Table 1:** Demographic information for participants.

	Number (%)
Total respondents	n = 112 (100%)
Gender - Female	94 (83.9%)
Age	
20-29 years	58 (51.8%)
30-39 years	31 (27.7%)
> 40 years	23 (20.5%)
Years of clinical practice	
0- 4 years	39 (34.8%)
5- 9 years	32 (28.6%)
>10 years	41 (36.6%)
Clinical setting	
Hospital*	69 (61.6%)
Non Hospital **	43 (38.4%)
Position type	
Rotates to different clinical areas	52 (46.4%)
Static position	60 (53.6%)
Employment status	
Full time (≥38 hours/week)	92 (82.1%)
Part time	20 (17.9%)

* Hospital settings include: inpatient or outpatient

** Non hospital settings include: community outpatients, domiciliary patients, residential care settings, mental health (acute, community and extended care) and mixed settings

Responses from the qualitative sections of the survey (sections two, three and four) and the focus group discussions were analysed by two researchers who coded responses and grouped them into categories independently of one another, before meeting to reach a consensus about any differing categories. These categories were aligned under themes (see Table 2 for themes and categories). Within each theme, the prominent emerging category or categories were identified based on the frequency and nature of responses (with many similar or related responses indicating a primary emerging category). These prominent categories for strategies currently in use and for strategies that could have potential future use are discussed in the text of the results section (in addition to Table 2). Non-prominent categories are listed in Table 2, but are not discussed in the results text. A third independent researcher was available to mediate any coding disagreement between the two primary coders; however, no such disagreement occurred. When a single survey response contained multiple strategies that aligned with one or more themes, each relevant component was coded separately and grouped appropriately. Survey data was analysed by discipline and collectively to identify if any differences existed in strategies suggested by discipline (PT versus OT). However, there was no differentiation between disciplines in the analysis of focus group data (as members of both professional disciplines participated in each focus group). Example quotes from each theme are displayed in Table 3 regarding current and potential strategies to reduce WRMD.

**Table 2:** Perceived strategies (current and potential) to reduce work related musculoskeletal disorders amongst physiotherapists and occupational therapists.

Departmental or Organisational	Work load & work allocation	Work Practices	Work Environment & Equipment	Physical Condition or Capacity	Education and Training
*Factors relating to how a department or organisation is run*	*Factors relating to how the work is distributed & how staff manage their workload*s	*Factors relating to how the work is actually performed*	*Factors relating to the physical environment and resources*	*Factors relating to an individual's physical capacity & what is required to maintain this*	*Factors relating to education and training*
1. Rotational positions	1. Taking sufficient breaks	1. Use of therapy assistants	1. Availability of equipment	1. Own physical fitness & condition	1. Manual handling training
2. Appropriate staffing levels	2. Management of workload	2. Modification of treatment techniques	2. Having appropriate equipment	2. Access to health & fitness facilities	2. General competencies & training
3. Appropriate workloads	3. Appropriate workload	3. Applying ergonomic principles	3. Modifying clinical environment or setup	3. Work based fitness & conditioning	3. Professional development
4. Encourage staff health & fitness	4. Mixed patient caseload	4. Use of equipment	4. Modifying work station setup	4. Capacity to self manage	4. Training in use of equipment
5. Availability of staffing	5. Inclusion of non clinical tasks	5. Modification of treatment approach	5. Storage of equipment	5. Staff exercise classes	
6. Provision of Injury management strategies	6. Sharing of workloads	6. Avoiding particular tasks or activities	6. Maintenance of equipment	6. Early treatment	
7. Opportunities for change of role	7. Pacing of work activities	7. Application of manual handling skills	7. Mechanisms to transport equipment	7. Private physiotherapy treatment	
8. Funding & resources		8. Assistance of other therapists	8. Consultation for equipment purchase & acquisition	8. Fit for job assessments	
9. Culture of injury reporting culture			9. Appropriate vehicles		
10. Appropriate employer & consumer expectations					
11. Part time positions					
12. Sick leave cover					
13. Mixed/split rotations					
14. Management of rostered overtime					
15. Team building					

**Table 3:** Example participant quotes aligned under six themes regarding current or potential strategies to prevent work related musculoskeletal disorders.

Departmental or Organisational	Work load & work allocation	Work Practices	Work Environment & Equipment	Physical Condition or Capacity	Education and Training
*Factors relating to how a department or organisation is run*	*Factors relating to how the work is distributed & how staff manage their workload*s	*Factors relating to how the work is actually performed*	*Factors relating to the physical environment and resources*	*Factors relating to an individual's physical capacity & what is required to maintain this*	*Factors relating to education and training*
"Being able to rotate from a heavier to a lighter work area..."	"...reduce caseloads to reduce incidence of performing tasks with a poor technique due to inadequate time."	"...vigilant in using good body mechanics."	"...appropriate equipment used appropriately."	"...identify pain or discomfort early and seek treatment"	"...staff educator to provide 'expert' advice for handling of complex patients."
"Various project roles give me a break form patient contact."	"...avoid allocating many similar patient types and activities to one person."	"...modified manual techniques to reduce stress on thumbs & wrists."	"Spend more time setting up environment..."	"Annual fitness assessments for physically demanding roles..."	"Ensure adequate training of new staff..."
"...encourage reporting of aches and pains"	"...workload is often too large that breaks get missed regularly."	"Use of a second person to help with patient handling..."	"We need more space around beds so you do not have to put body in obscure positions to manoeuvre around furniture and attachments (IV poles etc.)"	"Mandatory physical fitness activities during work time..."	"Update of knowledge on how to use equipment appropriately..."
"...being able to work reduced hours by one day per week"	"Reduce the amount of time doing repetitive tasks..."	"...avoid the activity..."	".Maintaining equipment in optimal working order..."	"...regular holidays to give me a break!"	"...to assist in developing specialised skills and techniques to perform tasks required."
"...ensure therapists aren't forced to work overtime due to unrealistic heavy caseloads."	"Allow time for non clinical tasks to balance out clinical tasks..."	"...make clients use more self management approaches to rehabilitation."	"Provision of station wagons for carrying equipment on home visits..."	"...exercise regularly to maintain strength and flexibility."	"Training in joint protection strategies..."

### Ethics

This investigation conformed to the Declaration of Helsinki and local legislation. The investigation was undertaken within the bounds of the Princess Alexandra Hospital local ethical guidelines. Under local requirements, full organisational HREC review was not required (on the basis that the investigation did not involve patient participants and staff participation was considered to carry negligible risk). Instead, project approval and approval for staff participation was granted by the organisation executive and associated department directors who endorsed the project. Staff participation was voluntary. Staff gave informed consent for participation.

## Results

### Demographics

Overall 112 (34.3%) of the invited health professionals completed the qualitative survey of which 66 (58.9%) were PT and 46 (41.1%) were OT). All respondents chose to complete the survey online (none completed and returned a hardcopy of the survey). Demographic information for survey participants is displayed in Table 1. Seventy five (66.9%) respondents reported that they considered their current workload to be physically demanding.

### Risk Factors

The risk factors most frequently perceived by health professionals as likely to limit their capacity to continue working in physically demanding roles were: work postures and movements, lifting or carrying, patient related factors and repetitive tasks.

Work postures and movements included: working in the same posture for long periods, bending or twisting, reaching and working away from your body. Lifting and carrying included: carrying, lifting or moving heavy materials or equipment as well as lifting or transferring dependent patients. Patient related factors included: unanticipated sudden movements or fall by patient, working with bariatric patients, and patients with attachments and equipment. Repetitive tasks included: performing the same task repeatedly and treating large numbers of patients in one day.

### Strategies

Current and potential strategies to enable health professionals to continue working in physically demanding roles were grouped into six themes (see Table 2).

#### Theme 1: Organisational strategies

The primary emerging strategy reported in the organisational theme related to opportunities for staff to work in positions which rotate through different clinical areas. Physiotherapists reported using these opportunities to move to less physically demanding clinical roles. Moving to non-clinical roles including project officer or research positions, senior or management roles reduced physical demands. Swapping or giving away rostered overtime and on-call shifts was a prominent currently used strategy.

There were more responses for potential or future strategies in this theme than any other theme. The most frequent emerging strategies included maintaining appropriate staffing levels and ensuring existing positions were recruited to and filled. Other suggestions focused on ensuring appropriate workloads and aligning organisation and consumer expectations in relation to appropriate health professional workloads. Staffing to cover sick leave and rostered days off were also identified as a specific need to reduce additional demand of covering an absentee's workload. It was also suggested that organisations should take an active role in encouraging staff to maintain good general health and fitness.

Developing a culture that facilitated the reporting and effective management of WRMD and the provision of injury management strategies such as *'priority access to physiotherapy or a staff clinic to provide advice and treatment for work related musculoskeletal disorders' *were also suggested as potential strategies.

#### Theme 2: Workload or work allocation

Individual staff member's management of their workload including patient scheduling and variation of work tasks throughout a day was the most commonly reported strategy in this theme. However, it was also reported that this is not always possible due to limitations on availability of patients and other staff such as therapy assistants or language interpreters. Other strategies reported included interspersing non clinical tasks into workloads and controlling mix of patient types (both conditions and level of impairment).

The key issue raised by focus groups was the large number of patients requiring treatment on any given day. Health professionals stated that time pressures commonly resulted in staff *"cutting corners" *while rushing to complete tasks, potentially compromising quality of care and safety for the patient, the staff member or both. In line with survey responses, staff in focus groups reported not taking their break entitlements due to workload pressures. Participants expressed a desire to be able to say *'no' *to treating patients of a lower priority when workloads exceed capacity and for this be supported by management.

#### Theme 3: Work Practices

Respondents frequently reported modification of treatment techniques to reduce physical demands. To do this PT reported applying ergonomic principles, use manual handling skills, and avoiding particular work tasks whilst OT reported use of equipment.

The work practices theme had the fewest number responses for potential strategies. The use of therapy assistants was reported as a current strategy, but was also the most commonly suggested potential strategy. Focus groups suggested formal ergonomic assessment for specific work tasks and practices, for example manually assisting patients to maintain knee extension during gait training, and using this information to develop guidelines for safe clinical practice.

#### Theme 4: Work Environment and Equipment

Work environment and equipment were considered by focus group participants to contribute to the demand and risk associated with work tasks. Availability of equipment was the most frequently reported strategy in this theme, particularly amongst PT. Although use of equipment is a current strategy, health professionals expressed frustration with equipment limited in application by either being "*inappropriate" *or containing various design flaws. Health professionals felt that they should have a greater role in consultancy for equipment purchases. A need to improve access to equipment which is often stored in "*overcrowded" *storage areas or in locations distant to clinical areas was identified. It was also reported that inadequate maintenance of equipment resulted in additional physical demand on the treating health professionals.

Focus group respondents reported modifying both clinical environments and administrative work areas. Existing administrative work areas were often considered problematic due to limited space, poor location and set up of workstations, and large number of users accessing limited workstations within health facilities.

Future strategies within this theme focused on provision of equipment suitable for its intended task or purpose and compatible with other equipment items, as well as sufficient quantities of equipment. Additional potential strategies were improvements in the location, storage and transport of equipment. Occupational therapists suggested improving methods of transporting equipment with consideration of the size, shape and weight of equipment and the number of items needing to be transported at one time. A number of suggestions were made in focus groups regarding home visits, including availability of appropriate vehicles for home visits, *'parking located close to office*(s)*' *and patients, and appropriate transportation of equipment.

#### Theme 5: Physical Condition and Capacity

Health professionals' maintenance of their own physical fitness and condition to undertake work tasks was the most prominent strategy in this theme. Responses had a focus on potential corporate strategies to facilitate health and fitness of staff with some respondents suggesting this should be mandatory and preformed during work time. Other suggestions included fit for job assessments such as an *'annual fitness assessments for those in physically demanding roles' *and musculoskeletal screening of staff working in *'high risk' *clinical areas.

#### Theme 6: Education and training

Responses for current and future strategies in this theme focused on training and competencies, including existing *'manual handling' *training. Suggested potential strategies in this theme were for education on topics including injury prevention and sustainable work practices in physically demanding roles. The suggestions included '*training in alternative handling and postures'*, gaining knowledge of *'less physically demanding' *yet effective treatment techniques, manual handling training specific to clinical settings in addition to generic organisation wide training, and education and training regarding the use and application of equipment.

#### Summary of most prominent strategies

Overall the most reported current strategy used by health professionals to enable them to continue physically demanding roles was maintaining an appropriate level of physical fitness and condition. Following this was the use of therapy assistants, and working in positions that rotate periodically through a variety of clinical areas. The modification of treatment techniques and workload management strategies were the next most commonly reported strategies. Differences between health disciplines included a greater focus on the use of equipment by OT. PTs' responses had more emphasis on the use of therapy assistants, application of manual handling skills and ergonomic principles, modification of treatment techniques and avoidance of particular activities or tasks.

The most suggested potential strategies across all themes were appropriate staffing levels (including both health professionals and assistant staff), appropriate equipment, appropriate workloads, access to health and fitness facilities.

## Discussion

### Main Finding

This investigation has revealed that the factors perceived by participating health professionals as most likely to limit their capacity to continue working in a physically demanding role are work postures and movements, lifting or carrying, patient related factors and repetitive tasks. These findings are consistent with previous studies [4,5,8,9,19,26,28]. It is also noteworthy that some of these risk factors are inherent to the nature of clinical work for OT and PT professionals [19]. Although it may be possible to utilise strategies to reduce some of these risk factors, it would be unrealistic to expect a complete abolition of risk.

Survey and focus group data revealed a spectrum of current and potential strategies to enable health professionals to continue to work in physically demanding roles. The strategies were coded into six themes including organisational strategies, workload or work allocation, work practices, work environment and equipment, physical condition and capacity, and education and training (Table 2) and have been described in detail.

These themes represent a much broader range of classification than those presented in previous studies[5,8,9] which focused on use of aids and self protective strategies such as modification of technique or the environment. Health professionals presented a greater diversity of strategies in this study than those previously reported [5,8,9]. The strategies reported in this investigation are consistent with legislative requirements[23] and policy documents from relevant professional bodies[24,25] and are worthy of consideration by all stakeholders. The results from this investigation may assist future efforts to reduce WRMD and are necessary to reduce the impact of WRMD for individuals and organisations. Reducing the impact of these conditions has potential to improve job satisfaction, career longevity, staff retention and maintain a larger viable workforce.

The most frequently reported strategies currently used, with the exception of rotational positions, were directly related to the most commonly perceived risk factors. Many of the strategies reported were ones the respondents had a direct capacity to implement individually. These were consistent with the strategies suggested in previous studies [5,8]. The less frequent reporting of organisational or departmental strategies may not necessarily be an indictment on the institutions involved in this investigation. Rather it may reflect acuity in self awareness (in comparison to organisational awareness) in relation to strategies that are already in use. Nonetheless, the large number of potential organisational strategies suggested may indicate that respondents perceived there was a great deal more that could be done from an organisational perspective to prevent WRMD. This was also consistent with many of the potential strategies being dependent on funding and or resource allocation at an organisation level. Given organisational responsibility to assess and manage risk, greater investment in strategies to effectively prevent and manage WRMD may be warranted. Further investigation along this line is justified and should consider impact on patient care, health practitioners, workforce capacity and staff retention.

There was a wide range of strategies reported to have been used by respondents, yet the incidence of injury remains high amongst these professional groups. On one hand, this may indicate that the suggested strategies are either ineffective or inadequate to deal with the degree of risk and magnitude of the problem. Alternatively, the strategies may be utilised inconsistently or sub-optimally. For example, therapy assistants may not always be available to assist with physically demanding treatments, or physically demanding treatments are delivered consecutively whilst a therapy assistant is available. Similarly, maintaining a high level of physical fitness and condition may be a good strategy, but not all respondents may have the desire or opportunity to maintain high levels of physical fitness. Additionally, some strategies are beyond control of the average clinician, such as staffing and workload allocation, Responses indicate that current staffing levels are frequently perceived to be inadequate and workloads too high. From this investigation alone it is not possible to determine whether these claims are justified. However, given the frequency and consistent nature of responses on this topic, further inquiry regarding appropriate workload and staffing levels among these professional groups is warranted.

Extensive investigation into the prevention of WRMD amongst nurses has shown that multi-modal or multidimensional strategies can be effective in reducing WRMD [6,7,18]. Despite differences in clinical tasks it is likely that the same may be true for PT and OT professionals given the similarities in clinical environment and patient types. Seven strategies reported in previous multidimensional interventions to reduce WRMD associated with patient handling include: equipment provision/purchase, education and training, risk assessment, review and change of policies and procedures, change or introduction of patient assessment system, work environment redesign, and work organisation/practice change [18]. The strategies reported in this study represent similar dimensions, indicating that similar multidimensional interventions tailored to PT and OT professionals are worthy of investigation.

Work related musculoskeletal disorders pose a significant threat to the career path and longevity of healthcare professionals, and to maintenance of a viable workforce for healthcare organisations. Health professionals have implemented measures in attempts to prevent injury and enable themselves to continue working in physically demanding roles. Many of the additional strategies that could be implemented are dependent on resource or procedural changes at an organisational level. Complete elimination of risks may not be possible due to the nature of clinical tasks, however, from the number and variety of potential strategies suggested by health professionals it is evident that more could be done to prevent injuries amongst health professionals working in physically demanding roles.

#### Strengths and limitations

The response rate (34.3%) was not high. This may be attributable to relatively short data collection period (3 weeks) and primarily non face-to-face invitation to participate (initial email contact). Nonetheless for an exploratory qualitative investigation of this nature the sample (n = 112) was sufficient to address the research aims. However, the ability to extrapolate results from this investigation to other health professions is limited by the sample consisting entirely of two professional groups. Furthermore the respondents were all working in public healthcare at the time of the investigation.

Other attributes of the sample were valuable to this investigation. The sample contained representation from professionals across the spectrum of healthcare settings which ranged from acute wards in tertiary hospitals to outpatient and domiciliary based settings. This investigation also included a substantial proportion (34.8%) of professionals in their first five years of clinical work, the timeframe in which the majority[5] and often most serious [16] WRMD occur. Additionally, greater than one third of the sample had more than 10 years experience. Based on Bork's[4] assertion of survivor bias this is an important population to consider, as 'more experienced' therapists are those who are likely to have developed strategies to enable them to cope with the physical demands of the work. Hence the mix of settings and experience is likely to have contributed to a completeness of responses. Further limitation exists in the qualitative study design and analysis which did not allow for assertions to be made regarding the effectiveness of the strategies reported. However, the purpose of the study was exploratory in nature seeking only to investigate these health professionals' perceived risks, current and potential strategies rather than the effectiveness of these strategies.

### Future research

The high levels of WRMD reported amongst health professionals working in physically demanding roles indicate that strategies to reduce the risk of injury are worthy of further investigation [4,22,23]. It would also be valuable for future research to investigate the specific nature of physical demands on PT and OT clinicians working in various clinical settings to allow the tailoring of WRMD prevention strategies. The strategies presented in this study require further investigation to evaluate the comparative effectiveness of the particular strategies in relation to others. Given the strategies were suggested by health professionals trained in prevention and management musculoskeletal disorders and who understand the demands of their clinical roles, the rationale for further investigation and implementation of the strategies reported in this investigation is credible.

## Conclusions

This investigation has revealed that work postures and movements, lifting or carrying, patient related factors and repetitive tasks are perceived as key risk factors for continuing to work in physically demanding clinical roles. Six themes for strategies to overcome these risk factors include organisational strategies, workload or work allocation, work practices, work environment and equipment, physical conditional and capacity, and education and training. Further investigation regarding the relative effectiveness of the identified strategies is warranted.

## Competing interests

The authors declare that they have no competing interests.

## Authors' contributions

LP contributed to research idea conception, data collection, data analysis and manuscript preparation, as well as manuscript review, appraisal and editing. SM contributed to research idea conception, data analysis and manuscript preparation, as well as manuscript review, appraisal and editing. Both authors read and approved the final manuscript.

## Pre-publication history

The pre-publication history for this paper can be accessed here:

http://www.biomedcentral.com/1471-2474/12/24/prepub
